# Supplementary material for: Multi-Omic Profiling, Structural Characterization, and Potent Inhibitor Screening of Evasion-Related Proteins of a Parasitic Nematode, Haemonchus contortus, Surviving Vaccine Treatment
Source: Biomedicines. 2023 Jan 30;11(2):411. doi: 10.3390/biomedicines11020411 (PMC9952990; doi:10.3390/biomedicines11020411)
Supplement: Supplementary file 1 [file biomedicines-11-00411-s001.zip › biomedicines-2109779 figure S1.pdf]

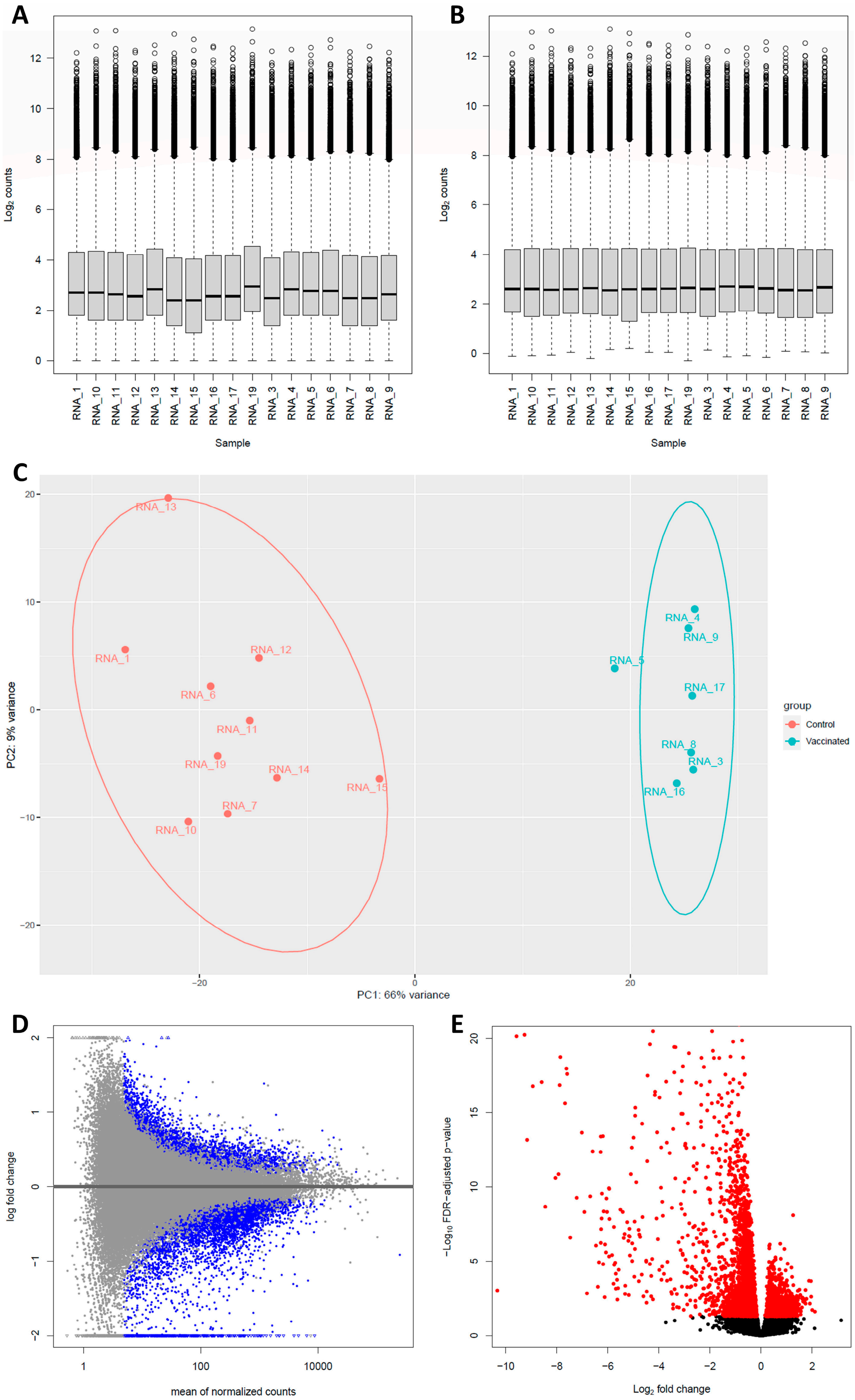
A
B
C
D
E
Log₂ counts
Sample
RNA_1
RNA_10
RNA_11
RNA_12
RNA_13
RNA_14
RNA_15
RNA_16
RNA_17
RNA_19
RNA_3
RNA_4
RNA_5
RNA_6
RNA_7
RNA_8
RNA_9
PC1: 66% variance
PC2: 9% variance
group
Control
Vaccinated
log fold change
mean of normalized counts
−Log₁₀ FDR−adjusted p−value
Log₂ fold change

Figure S1: Statistics and sample grouping of *H. contortus* adult males exposed to the vaccine response, or the control group's de novo transcriptome assemblies. Raw counts (A) were normalized (B) by a variance stabilizing transformation (D), and profiles are outlined using principal component analyses (PCA) of the normalized transcript counts measured in worms collected from vaccinated or control sheep (C). Volcano plot of significantly differentially expressed (DE) genes, determined using DESeq2 (E).
